# Summary data‐based Mendelian randomization and single‐cell RNA sequencing analyses identify immune associations with low‐level LGALS9 in sepsis

**DOI:** 10.1111/jcmm.18559

**Published:** 2024-07-23

**Authors:** Yongsan Yang, Lei Dong, Yanguo Li, Ye Huang, Xiaoxi Zeng

**Affiliations:** ^1^ Intensive Care Unit and West China Biomedical Big Data Center West China Hospital, Sichuan University Chengdu China; ^2^ Med‐X Center for Informatics Sichuan University Chengdu China; ^3^ Key Laboratory of RNA Biology, Center for Big Data Research in Health, Institute of Biophysics, Chinese Academy of Sciences Beijing China; ^4^ Institute of Drug Discovery Technology, Ningbo University Ningbo China; ^5^ Department of Emergency Medicine Xiyuan Hospital of China Academy of Chinese Medical Sciences Beijing China; ^6^ West China Biomedical Big Data Center West China Hospital, Sichuan University Chengdu China

**Keywords:** LGALS9, monocytes, single‐cell RNA‐seq, Summary data‐based Mendelian randomization, T cells

## Abstract

Sepsis is one of the major challenges in intensive care units, characterized by the complexity of the host immune status. To gain a deeper understanding of the pathogenesis of sepsis, it is crucial to study the phenotypic changes in immune cells and their underlying molecular mechanisms. We conducted Summary data‐based Mendelian randomization analysis by integrating genome‐wide association studies data for sepsis with expression quantitative trait locus data, revealing a significant decrease in the expression levels of 17 biomarkers in sepsis patients. Furthermore, based on single‐cell RNA sequencing data, we elucidated potential molecular mechanisms at single‐cell resolution and identified that LGALS9 inhibition in sepsis patients leads to the activation and differentiation of monocyte and T‐cell subtypes. These findings are expected to assist researchers in gaining a more in‐depth understanding of the immune dysregulation in sepsis.

## 1 INTRODUCTION

Sepsis, characterized by the host inflammatory response triggered by severe, life‐threatening infection and accompanied by organ dysfunction,^1^ represents the most formidable challenge in intensive care units.^2^ And immunosuppression is increasingly acknowledged as a primary contributor to mortality in sepsis patients. The immunosuppressive state induced by sepsis arises from the perturbation of immune homeostasis.^3^ For example, T‐cell apoptosis may lead to organ failure, tissue damage and immunodeficiency.^4^ On the other hand, markers of the immunosuppressive phase of sepsis, such as anti‐inflammatory cytokines, and alterations in the cell surface markers of monocytes and lymphocytes, have played an important role in sepsis.^5^ Therefore, there may be a relationship between these biomarkers and the disruption of immune homeostasis, underscoring their significance in the treatment and prognosis of sepsis.

The colocalization between disease genome‐wide association studies (GWAS) and expression quantitative trait locus (eQTL) signals has been utilized as a fine‐mapping approach to successfully identify candidate causal variants and candidate causal genes at disease risk loci.^6^ Previous studies have shown that the Summary data‐based Mendelian randomization analysis (SMR) method^7^ can help us identify target genes causally related to the disease. For instance, Krishnamoorthy et al. identified 38 novel genes associated with severe COVID‐19 using the SMR method, providing insights for the development of therapeutic agents for treating severe COVID‐19.^8^ SMR is a method to integrate aggregate statistics from GWAS and eQTL data within the Mendelian randomization (MR) framework. This method helps to identify genes whose expression levels are linked to complex traits. While conventional two‐sample MR utilizes summary statistics from two independent GWAS to estimate the effect of one phenotype on another, SMR utilizes summary statistics from independent eQTL studies and GWAS to estimate the effect of a gene's expression level on a phenotype.

While these studies have provided insights at the phenotype level, a more in‐depth elucidation of the detailed pathogenic mechanisms of the risk factors is still required. Single‐cell RNA sequencing (scRNA‐seq) technology is a powerful tool for analysing microscopic mechanisms at the single‐cell level and has been widely applied in research on various diseases, including sepsis.^9^, ^10^ In this study, we first conducted SMR analysis by integrating summary data from GWAS and eQTL studies to estimate the causal impact of sepsis at the gene level. Subsequently, we performed scRNA‐seq analysis on a sepsis dataset, uncovering potential mechanisms that significantly suppress LGALS9 expression in sepsis. Our findings are significant for a better understanding of the relationship between sepsis and immune homeostasis, and may provide guidance for the treatment of sepsis.

## 2 METHODS

### 2.1 SMR analysis

#### 2.1.1 Data collection

**FIGURE 1:**
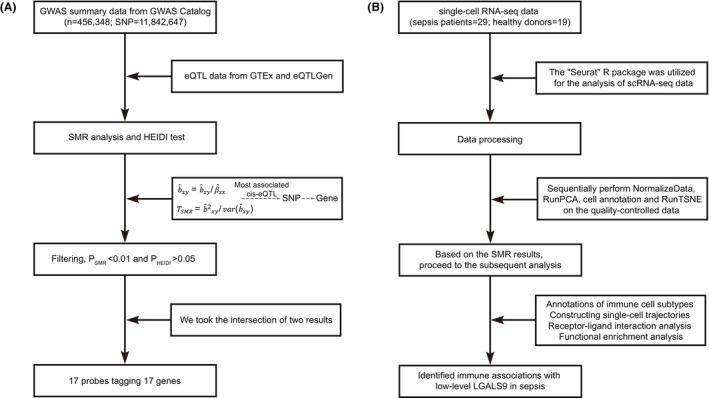
Workflow diagram. Flow chart for the (A) SMR analysis and (B) scRNA‐seq analysis.

In this study, we utilized eQTL data from two sources (Figure 1A). We obtained whole blood sample data from the Genotype‐Tissue Expression (GTEx) project,^11^ and blood sample data from the eQTLGen Consortium.^12^ The GWAS summary data for sepsis were derived from the work of Jiang et al.,^13^ which involved 456,348 European samples and 11,842,647 SNPs, as documented in the GWAS catalogue (study accession: GCST90044692).^14^

#### 2.1.2 Causal impact of gene expression on sepsis

We conducted a meticulous SMR analysis utilizing the SMR software (version 1.3.1), employing eQTL as the instrumental variable, gene expression as the exposure variable and sepsis as the outcome. The specific formula is as follows:

$${\hat{b}}_{\mathrm{xy}}={\hat{b}}_{\mathrm{zy}}/{\hat{\beta }}_{\mathrm{zx}}$$

In this formula, *x* represents the expression level of a gene, *y* represents the trait and *z* represents a genetic variant. ${\hat{b}}_{\mathrm{xy}}$ represents the effect size of gene expression on the trait. ${\hat{b}}_{\mathrm{zy}}$ represents the estimate of the SNP effect from a GWAS for the trait, and ${\hat{\beta }}_{\mathrm{zx}}$ represents the estimate of the SNP effect on the expression level of the gene from an independent eQTL study. Then the statistic $T_{\mathrm{SMR}}$ was calculated as follows:

$$T_{\mathrm{SMR}}={{\hat{b}}^{2}}_{\mathrm{xy}}/\mathrm{var}\left({\hat{b}}_{\mathrm{xy}}\right)$$

Furthermore, we performed the Heterogeneity in Dependent Instruments (HEIDI) test to assess the presence of linkage in the observed association. HEIDI utilizes multiple SNPs within the eQTL region to differentiate between pleiotropy and linkage effects.

### 2.2 scRNA‐seq analysis

#### 2.2.1 Data processing

The single‐cell RNA‐seq data for 29 sepsis patients and 19 healthy donors were obtained from Reyes et al.^15^ (Figure 1B). The datasets were stored in the Broad Institute Single Cell Portal database (SCP548), and peripheral blood sample data were selected for downstream analysis in our study. The “Seurat” R package was utilized for the analysis of scRNA‐seq data. Initially, we employed the *scDblFinder* function to pinpoint doublets within the integrated dataset. Subsequently, we performed quality control on cells, considering indicators such as the total number of RNA molecules per cell (500 < *n*Count_RNA < 3000), the number of features per cell (1000 < *n*Feature_RNA < 10,000) and the proportion of mitochondrial genes per cell (percent.mt <5), and ensured that the *scDblFinder.class* was labelled as ‘singlet’, to retain high‐quality cells for downstream analysis. In addition, 83,210 cells were ultimately obtained. Second, the raw RNA count was normalized using the *NormalizeData* function. Then, the cells were clustered by principal component analysis (PCA) using the *RunPCA* function. We used this function to perform PCA dimensionality reduction using 2000 highly variable genes and the top 15 principal components. In addition, we also utilized the Harmony^16^ method to remove batch effects from the scRNA‐seq data. Finally, the cells were annotated using marker genes from published papers, and then t‐SNE projection visualization of the clusters was performed using the *RunTSNE* function.

#### 2.2.2 Annotations of immune cell subtypes

To identify cell subtypes within immune cells (T cells, Mono/Macrophages), we individually extracted and performed a second round of clustering on each of these cell types, following the same procedure as the first round. Subsequently, we annotated the clusters obtained from the re‐clustering process.

#### 2.2.3 Constructing single‐cell trajectories

Cellular trajectory analysis reconstructs cellular changes over time, enabling inference of cellular evolution and differentiation at the single‐cell level. To explore the differentiation and developmental trajectories of specific cell subtypes, we utilized the “monocle3” R package^17^, ^18^ for rigorous analysis.

#### 2.2.4 Receptor–ligand interaction analysis

To analyse the receptor–ligand pairing between different types of cells, we used CellChat,^19^ an R package designed for inference, analysis and visualization of cell–cell communication from single‐cell data. Moreover, we obtained protein interaction information from the STRING database.^20^

#### 2.2.5 Functional enrichment analysis of key genes

The DAVID database^21^ was employed for enrichment analysis of Gene Ontology (GO) Biological Processes (BP), Cellular Components (CC), Molecular Functions (MF) and KEGG pathways. We set the significance threshold at *p* < 0.05, and these GO terms and pathways were visualized using the “ggplot2” R package.

## 3 RESULTS

### 3.1 SMR analysis identified sepsis‐related gene targets

In order to identify regulatory genes that are causally linked to the onset and progression of sepsis disease, we utilized SMR to analyse the GWAS and eQTL data (see Section 2), applying a threshold of *P*_SMR_ <0.01 and *P*_HEIDI_ >0.05 to pinpoint significant probes. Moreover, we took the intersection of these two results for subsequent analysis. The results are shown in Table 1. There were 17 probes tagging 17 genes whose expressions were pleiotropically associated with sepsis. As reported in previous studies, LGALS9 (galectin‐9) exhibited a protective effect in polymicrobial sepsis,^22^ SIGLEC7 was an important negative regulator of acute inflammatory responses and was a potential target for the treatment of sepsis.^23^ Next, we examined the expression of these genes in single‐cell data to determine their associations with immune cells.

**TABLE 1:** Summary data‐based Mendelian randomization (SMR) analysis results.

No.	Probe	Gene	PSMR.GTEx	PHEIDI.GTEx	PSMR.eQTLGen	PHEIDI.eQTLGen
1	ENSG00000237803	LINC00211	0.009226	0.887229	0.008638	0.959078
2	ENSG00000186583	SPATC1	0.004655	0.877144	0.001026	0.76462
3	ENSG00000135945	REV1	0.005043	0.842616	0.003991	0.339622
4	ENSG00000224389	C4B	0.001855	0.053772	0.000147	0.794655
5	ENSG00000244731	C4A	0.000605	0.787618	0.0004	0.531305
6	ENSG00000103222	ABCC1	0.006316	0.766289	0.004455	0.90374
7	ENSG00000152219	ARL14EP	0.00708	0.746993	0.006272	0.459003
8	ENSG00000212283	SNORD89	0.002608	0.634934	0.002294	0.581523
9	ENSG00000172469	MANEA	0.000574	0.454086	0.000431	0.301327
10	ENSG00000168961	LGALS9	0.008532	0.450986	0.008228	0.419724
11	ENSG00000229314	ORM1	0.006999	0.411255	0.005649	0.396145
12	ENSG00000151655	ITIH2	0.006012	0.333064	0.000344	0.143914
13	ENSG00000189339	SLC35E2B	0.000395	0.323456	0.000385	0.075599
14	ENSG00000168995	SIGLEC7	0.005186	0.312433	0.00167	0.866999
15	ENSG00000146733	PSPH	0.000365	0.092842	0.000332	0.215259
16	ENSG00000228278	ORM2	0.008279	0.086495	0.005709	0.250908
17	ENSG00000197530	MIB2	0.007346	0.076134	0.003483	0.265407

### 3.2 Integrated analysis of the sepsis single‐cell transcriptional atlas

**FIGURE 2:**
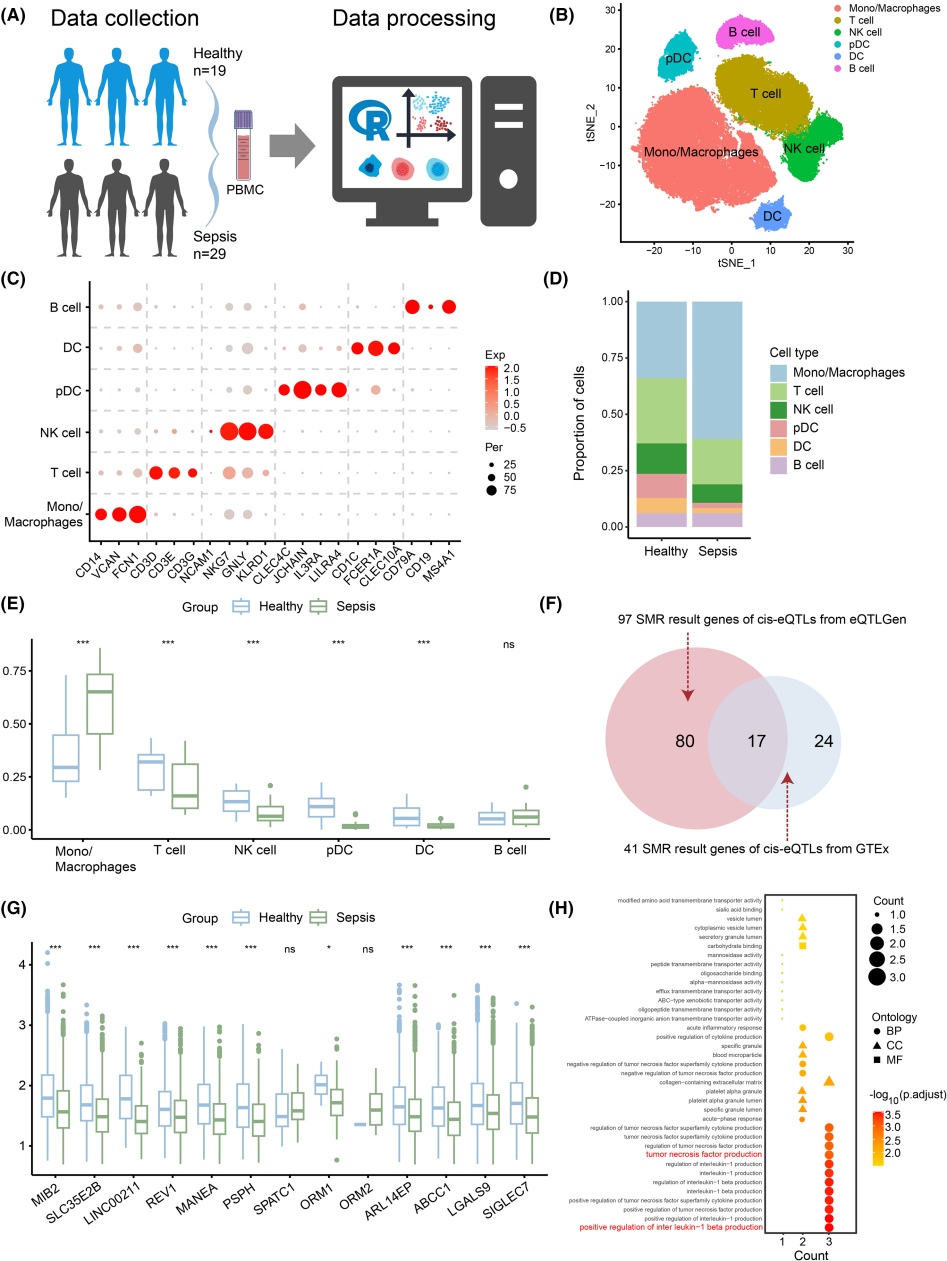
Identification of cell types by scRNA‐seq in sepsis patients. (A) Data collection and data processing for scRNA‐seq. (B) T‐distributed stochastic neighbour embedding (t‐SNE) visualization of the annotations for six immune cell types. (C) Bubble plot showing marker genes across six cell types, dot size indicates fraction of expressing cells, coloured according to expression levels. (D) Bar plot showing the contribution of each cell type in the Healthy group and Sepsis group. (E) Box plot showing the difference in the proportion of each cell type in the Healthy group and Sepsis group. (F) Venn diagram showing the overlap of SMR results from two sources of eQTL data and revealing that 17 genes are identical in both results. (G) Box plot showing the expression levels of the 17 genes in all cells of the Healthy group and Sepsis group. (H) GO term enrichment analysis of the 17 genes. Significance was determined by two‐sided Wilcoxon test in (E) and (G), ns, *p* > 0.05, *p < 0.05, **p < 0.01 and ***p < 0.001.

Our SMR analysis results have confirmed the causal relationship between these genes and sepsis. To further understand the role of these genes, we applied scRNA‐seq data to reveal the mechanisms promoting or inhibiting gene expression levels in sepsis. We constructed a single‐cell transcriptional atlas of peripheral blood samples obtained from 29 sepsis patients and 19 healthy donors as controls (Figure 2A). After rigorous quality controls, a total of 83,210 high‐quality cells were ultimately obtained (see Section 2). Finally, we identified 17 significant cell clusters and categorized them into six major cell types, including B lymphocytes (B cells), T lymphocytes (T cells), monocytes and macrophages (Mono/Macrophages), natural killer cells (NK cells), dendritic cells (DC) and plasmacytoid dendritic cells (pDC). All the cells were visualized based on t‐distributed stochastic neighbour embedding (t‐SNE) projection (Figure 2B). Among these cells, B cells were characterized by higher expression of genes including CD79A, CD19 and MS4A1. T cells expressed a unique set of marker genes including CD3D, CD3E and CD3G. Likewise, DC and pDC were clustered separately with different markers; for example, CD1C, FCER1A and CLEC10A were expressed specifically in DC, while CLEC4C, JCHAIN, IL3RA and LILRA4 were expressed specifically in pDC. CD14, VCAN and FCN1 were highly expressed in Mono/Macrophages, whereas NCAM1, NKG7, GNLY and KLRD1 were highly expressed in NK cells (Figure 2C). At the same time, we observed a significantly higher proportion of Mono/Macrophages in sepsis patients compared to the healthy controls, while the proportion of T cells in the healthy controls was significantly higher than that in sepsis patients (Figure 2D,E). These findings are consistent with the results of Yao et al. and Heffernan et al.^24^, ^25^ Our results indicated that the occurrence of sepsis leads to changes in the proportions of immune cell subsets in the human body, thereby affecting the dysregulation of immune function.

### 3.3 Expression levels of SMR outcome genes in sepsis patients

Next, we explored the expression of genes in the SMR analysis results in scRNA‐seq data, aiming to confirm the up‐ or down‐regulated genes in the two datasets (Figure 2F). The four genes C4A, C4B, SNORD89 and ITIH2 were not detected in the scRNA‐seq data, while the majority of the remaining genes showed significantly decreased expression in sepsis patients (Figure 2G). GO term enrichment analysis of these genes revealed significant enrichment in biological processes associated with inflammation. These include the positive regulation of interleukin‐1 beta (*p* = 2.83E‐06) and tumour necrosis factor production (*p* = 7.18E‐05), both of which play crucial roles in the pathophysiology of sepsis (Figure 2H, Material S1).

### 3.4 Activation and differentiation of Mono/Macrophages

The expression of these genes may disrupt the immune homeostasis of sepsis patients, and we further investigated the underlying mechanism in terms of cell subtypes. As is widely recognized, monocytes have been comprehensively characterized for their pivotal roles in lymphocyte activation, pathogen elimination and the facilitation of tissue repair via the secretion of inflammatory factors.^26^ Next, we focused on analysing the expression of these genes in subtypes of Mono/Macrophages.

**FIGURE 3:**
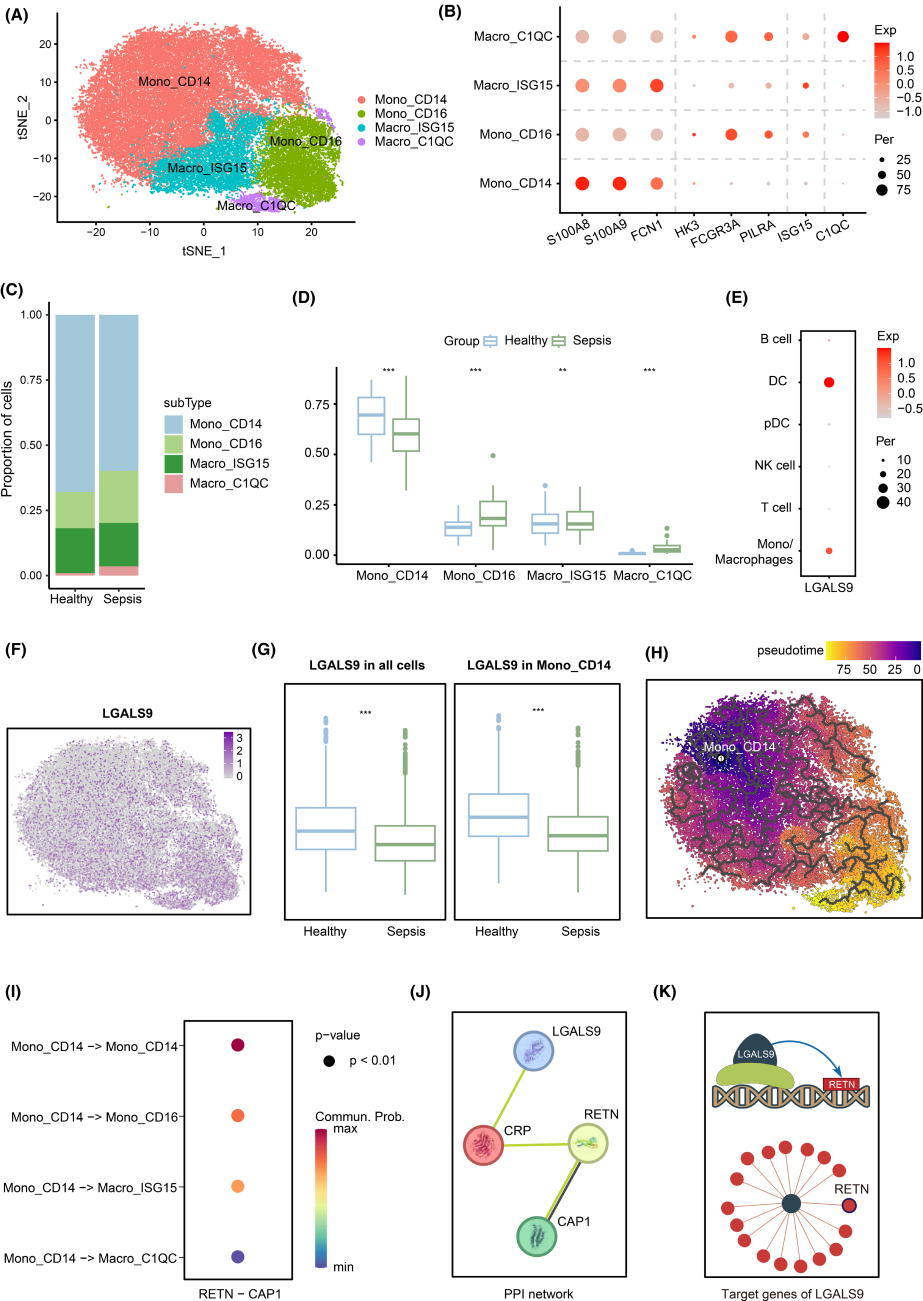
Characterization of Mono/Macrophages by scRNA‐seq in sepsis patients. (A) t‐SNE plot showing the major lineages of Mono/Macrophages. (B) Bubble plot showing marker genes across four cell subtypes, dot size indicates fraction of expressing cells, coloured according to expression levels. (C) Bar plot showing the contribution of each cell subtype in the Healthy group and Sepsis group. (D) Box plot showing the difference in the proportion of each cell subtype between the Healthy group and Sepsis group. (E) Bubble plot showing significant expression of LGALS9 in Mono/Macrophages. (F) Feature plot showing the expression level of LGALS9 in Mono/Macrophages. (G) Box plots showing the expression level of LGALS9 across all cells and CD14^+^ monocytes of the Healthy group and Sepsis group. (H) Results from cell trajectory analysis initiated with CD14^+^ monocytes as the initial cell population. (I) Bubble plot showing the results of cell–cell communication, dot colour reflects communication probability and dot size represents computed *p*‐value. (J) PPI network of LGALS9, RETN and CAP1 from the STRING database. (K) Network graph showing that RETN is one of the predicted target genes of the transcription cofactor LGALS9. Significance was determined by two‐sided Wilcoxon test in (D) and (G), **p < 0.01 and ***p < 0.001.

First, we further constructed an atlas of subtypes of Mono/Macrophages (see Section 2). The re‐clustering of Mono/Macrophages identified four cell subtypes: CD14^+^ monocytes (Mono_CD14), CD16^+^ monocytes (Mono_CD16), ISG15+ macrophages (Macro_ISG15) and C1QC+ macrophages (Macro_C1QC; Figure 3A). We revealed the gene signatures of all four subsets. Among these subsets, Mono_CD14 cells were characterized by high expression of S100A8, S100A9 and FCN1, while Mono_CD16 cells were characterized by high expression of HK3, FCGR3A (known as CD16A) and PILRA. Furthermore, Macro_ISG15 and Macro_C1QC cells were identified based on high expression of ISG15 and C1QC (Figure 3B). All of these marker genes were derived from the research conducted by Cheng et al.^27^ We then investigated the proportions of the four cell subtypes in samples and found that the proportion of Mono_CD14 cells in sepsis patients was significantly lower than that in the healthy controls, while the proportions of Mono_CD16, Macro_ISG15 and Macro_C1QC cells were significantly higher than those in the healthy controls (Figure 3C,D). In addition, we found that LGALS9, which is one of the genes identified in the SMR results, is highly expressed in Mono/Macrophages (Figure 3E). Subsequently, we examined the expression of LGALS9 across various subtypes and observed that LGALS9 is primarily expressed in Mono_CD16, Macro_ISG15 and Macro_C1QC cells. Notably, compared to healthy controls, the expression of LGALS9 in sepsis patients was significantly reduced, particularly within the Mono_CD14 subtype (Figure 3F,G).

We next aimed to investigate the impact of the low expression of LGALS9 on Mono_CD14 cells. Previous studies have reported that CD14^+^ monocytes could differentiate into CD16^+^ monocytes and macrophages in the inflammatory response.^28^, ^29^, ^30^, ^31^ We conducted a pseudotime analysis to investigate the developmental trajectories of these cells (see Section 2). The results revealed that the pseudotime of Mono_CD14 cells precedes that of Mono_CD16, Macro_ISG15 and Macro_C1QC cells (Figure 3H). We then calculated the attraction strengths of receptor–ligand pairs in scRNA‐seq dataset by CellChat (see Section 2), and found prominent RETN−CAP1 interaction between these cell subtypes (Figure 3I). Both RETN and CAP1 are associated with inflammation, and in sepsis patients, the intensity of RETN–CAP1 interaction increases, which is consistent with the research results of Sun et al.^32^ And then, we utilized the STRING database to construct a protein–protein interaction (PPI) network and identified the connections among these hub genes (LGALS9, RETN and CAP1; Figure 3J). Furthermore, we have also verified in AnimalTFDB4^33^ and TcoFbase^34^ databases that LGALS9 is a transcriptional cofactor, and both ARACNe^35^ and GENIE3^36^ methods have predicted that RETN is one of its target genes (Figure 3K, Material S1). Based on the above results, we speculate that the decreased expression of LGALS9 in sepsis patients will promote the activation and differentiation of CD14^+^ monocytes, leading to their differentiation into CD16^+^ monocytes and macrophages with stronger phagocytic and antigen‐presenting abilities during the inflammatory response.

### 3.5 Activation and differentiation of T cells

**FIGURE 4:**
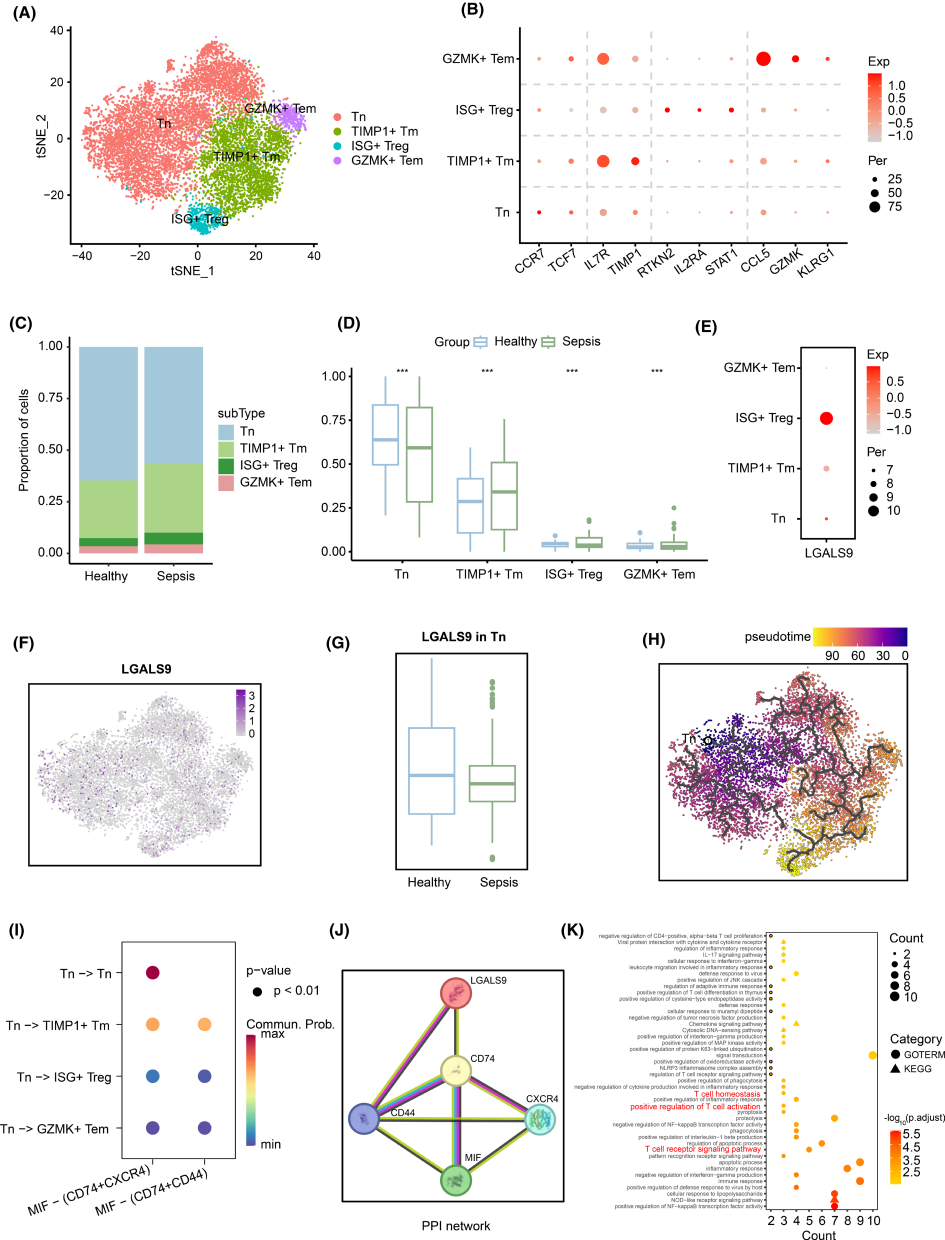
Characterization of T cells by scRNA‐seq in sepsis patients. (A) t‐SNE plot showing the major lineages of CD4^+^ T cells. (B) Bubble plot showing marker genes across four cell subtypes, dot size indicates fraction of expressing cells, coloured according to expression levels. (C) Bar plot showing the contribution of each cell subtype in the Healthy group and Sepsis group. (D) Box plot showing the difference in the proportion of each cell subtype between the Healthy group and Sepsis group. (E) Bubble plot showing significant expression of LGALS9 in CD4^+^‐naive T cells. (F) Feature plot showing the expression level of LGALS9 in CD4^+^ T cells. (G) Box plot showing the expression level of LGALS9 across CD4^+^‐naive T cells of the Healthy group and Sepsis group. (H) Results from cell trajectory analysis initiated with CD4^+^‐naive T cells as the initial cell population. (I) Bubble plot showing the results of cell–cell communication, dot colour reflects communication probability and dot size represents computed *p*‐value. (J) PPI network of LGALS9, CD74, CD44, CXCR4 and MIF from the STRING database. (K) GO term and KEGG pathway enrichment analysis of LGALS9 target genes. Significance was determined by two‐sided Wilcoxon test in (D), ***p < 0.001.

Interestingly, we also observed similar results in T cells. Here, we divided T cells into CD4^+^ and CD8^+^ subsets, and found differential expression of LGALS9 in CD4^+^ T cells. Similarly, we constructed an atlas of CD4^+^ T‐cell subtypes. The re‐clustering of CD4^+^ T cells identified four cell subtypes: naive T cells (Tn), TIMP1+ memory T cells (TIMP1+ Tm), GZMK+ effector memory T cells (GZMK+ Tem) and ISG+ regulatory T cells (ISG+ Treg) (Figure 4A). Among these subsets, Tn cells were characterized by high expression of CCR7 and TCF7. TIMP1+ Tm cells were characterized by high expression of IL7R and TIMP1, while GZMK+ Tem cells were characterized by high expression of CCL5, GZMK and KLRG1. Furthermore, ISG+ Treg cells were identified based on high expression of RTKN2, IL2RA and STAT1 (Figure 4B). These marker genes came from another study by Zheng et al.^37^ Based on the proportions of the four cell subtypes in the samples, we found that the proportion of Tn cells in sepsis patients was significantly lower than that in the healthy controls, while the proportions of the other three cell subtypes were significantly higher than those in the healthy controls (Figure 4C,D). We also found that LGALS9 was expressed mainly in Tn and ISG+ Treg cells (Figure 4E,F). In the Tn subtype, compared to the healthy controls, there was a decreasing trend in the expression of LGALS9 in sepsis patients, although it was not statistically significant (Figure 4G).

We also conducted a pseudotime analysis to investigate the developmental trajectories of these cells. The results revealed that the pseudotime of Tn cells precedes that of the other three cell subtypes (Figure 4H). Then, we used CellChat to calculate the attraction strengths of receptor–ligand pairs, and found prominent “MIF − (CD74+CXCR4)” and “MIF − (CD74+CD44)” interactions in these cells (Figure 4I). Studies have shown that MIF recruits inflammatory cells by binding to receptors and is involved in the occurrence and development of sepsis.^38^ Then, we found the connections of these genes (LGALS9, CD74, CD44, CXCR4 and MIF) in the PPI network provided by the STRING database (Figure 4J). We conducted GO term and KEGG pathway enrichment analysis using the predicted target genes of LGALS9, and found significant enrichment of T‐cell‐related biological processes, such as T‐cell receptor signalling pathway (*p* = 1.16E‐03), positive regulation of T‐cell activation (*p* = 5.42E‐03) and T‐cell homeostasis (*p* = 6.73E‐03; Figure 4K, Material S1). In summary, we speculate that the decreased expression of LGALS9 in sepsis patients may enhance the activation and differentiation of CD4^+^‐naive T cells, leading to their differentiation into regulatory T cells with stronger immune response capabilities during inflammation.

## 4 DISCUSSION

Immunosuppression and imbalance in immune homeostasis significantly contribute to the high mortality rate in sepsis patients.^39^, ^40^ Clinical research has identified several biomarkers associated with sepsis development, such as interleukins and tumour necrosis factors.^41^ However, the relationships between these biomarkers and immune homeostasis remain unclear. Therefore, our research employed the SMR analysis method to identify significant causal relationships between sepsis and 17 biomarkers by combining GWAS and eQTL data. These biomarkers may play important roles in immune response and inflammation regulation, and potentially impact the onset and development of sepsis. In subsequent analyses, combined with scRNA‐seq data, we further found that the decrease in LGALS9 expression level in sepsis patients has an impact on immune homeostasis.

In contrast to the observational studies, SMR analysis can avoid the influence of confounding factors on the results and provide critical information on causality, assisting us in identifying genes associated with diseases. Single‐cell sequencing technology was used to analyse gene expression at the single‐cell level. Through scRNA‐seq, researchers can gain deep insights into the heterogeneity among different cell types, discover rare cell subpopulations, identify cell state transitions and understand cell‐to‐cell interactions. By combining these two analytical methods, we found that the low expression of LGALS9 in sepsis patients is associated with the activation and differentiation of CD14^+^ monocytes and CD4^+^‐naive T cells. This discovery reveals the potential importance of LGALS9 in immune regulation, providing a new direction for future research to explore the potential of LGALS9 as a therapeutic target or biomarker.

In addition, our research also has certain limitations. First, in the SMR analysis, to demonstrate that changes in expression lead to changes in phenotype, we need to obtain a large number of independent SNPs that affect the expression level of the same gene from a very large eQTL study. Second, the GWAS summary data utilized in our analyses were primarily composed of participants of European ancestry. As a result, the generalizability of our findings to other ethnicities may be limited. Third, in the single‐cell analysis, all samples were collected from peripheral blood. Thus, we cannot provide a detailed description of the differences in other tissues of sepsis patients. Moreover, our collected single‐cell data lacked a large number of neutrophils, which play an important role in the immune defence of the body. This may be due to the single‐cell capture technology. These limitations will be addressed in our future work.

## 5 CONCLUSIONS

In this study, by integrating the results of SMR analysis and single‐cell RNA‐seq analysis, we have revealed that LGALS9 is expressed at lower levels in sepsis patients compared to healthy controls, and that low expression of LGALS9 promotes the activation and differentiation of CD14^+^ monocytes. Additionally, we found that the activation and differentiation of CD4^+^‐naive T cells are also linked to low expression of LGALS9 in sepsis patients.

## AUTHOR CONTRIBUTIONS

**Yongsan Yang:** Formal analysis (lead); visualization (lead); writing – original draft (lead). **Lei Dong:** Data curation (equal). **Yanguo Li:** Investigation (equal); visualization (equal). **Ye Huang:** Supervision (equal); writing – review and editing (equal). **Xiaoxi Zeng:** Funding acquisition (lead); supervision (lead); writing – review and editing (lead).

## FUNDING INFORMATION

This study was funded by the National Key Research and Development Program of China (grant no. 2022YFC2504500); Science and Technology Program, Sichuan, China (grant no. 2023NSFSC1598); and Science and Technology Program, Chengdu, Sichuan, China (grant no. 2022‐YF05‐01538‐SN).

## CONFLICT OF INTEREST STATEMENT

The authors declare that they have no competing interests.

## Supporting information


Data S1.

## Data Availability

The analysis code to produce the major results presented in the paper can be accessed from https://github.com/yangyongsan‐bio/Sepsis‐SMR‐scRNA.
